# Ventilation in American Dwellings; with a Series of Diagrams Presenting Examples in Different Classes of Habitations

**Published:** 1858-11

**Authors:** 


ART. V.—*Ventilation in American Dwellings; with a Series of Diagrams presenting Examples in Different Classes of Habitations.* By DAVID BOSWELL REID, M.D., F.R.S.E., etc. etc. To which is added *An Introductory Outline of the Progress of Improvement in Ventilation.* By ELISHA HARRIS, M.D., Late Physician-in-Chief to the New York Quarantine Hospital, etc. 8vo. pp. 124. New York: Wiley and Halsted, 1858.

As a nation, we have as yet made comparatively little progress in this important branch of hygiene; but our attention has been awakened to its great influence upon human health, by several eminent medical men of this country. More especially has this been effected by Dr. Reid, than whom no one has more thoroughly and practically investigated ventilation as adapted to the wants of all classes of society. Having resided among us two years, he has made ample use of his observations, and now presents us with the fruits of his labors, in the form of the most complete and systematic treatise that has yet appeared. Dr. Reid's experience has been large, and for the past few years he has been engaged in the introduction of his system in the largest houses of Great Britain and the Continent, as well as directing his attention to the ventilation of vessels. He can, therefore, speak *ex cathedra*, and we may fully rely upon the many suggestions contained in the work before us. It is to be hoped that architects and builders, for whom it is more especially intended, will read and re-read it, and fully digest its contents, and thus add more to our health and comfort. Many persons imagine that when they have a fine draught of air through their houses, the requisite object has been attained; but in this lies their mistake. Air may be fresh but not pure, and the first consideration of builders should be to make the proper arrangements for the free ingress and egress of pure air. This, moreover, should not be confined to the building, but the air without should be equally as pure, and special means should be employed for destroying the effluvia arising from sewers and marshes, which are in themselves rife sources of malarious diseases, cholera, and yellow fever. These points, as well as the ventilation of sick chambers, are carefully considered in Dr. Reid's work, which we can confidently recommend both to the professional and to the unprofessional reader.

